# Implications of acute change in estimated Glomerular Filtration Rate (eGFR) for the effect of sodium-glucose cotransporter-2 inhibitors (SGLT-2i) on long-term endpoints

**DOI:** 10.1371/journal.pone.0347741

**Published:** 2026-04-29

**Authors:** Ransmond O. Berchie, Lesley A. Inker, Hiddo J. L. Heerspink, Ben Haaland, Tom Greene

**Affiliations:** 1 Division of Biostatistics, Department of Population Health Sciences, University of Utah, Salt Lake City, Utah, United States of America; 2 Division of Nephrology, Department of Medicine, Tufts Medical Center, Boston, Massachusetts, United States of America; 3 Department of Clinical Pharmacy and Pharmacology, University of Groningen, The Netherlands; Hospital Costa del Sol, SPAIN

## Abstract

In randomized trials, the primary analysis often estimates the average treatment effect on a clinical endpoint. Some treatments also lead to early changes in a biomarker that is prognostic for the clinical endpoint, prompting investigators to explore how these acute biomarker changes might inform the treatment’s effect on long-term clinical outcomes. A naive analysis that directly examines treatment-by-biomarker-change interactions may lead to biased estimates because it fails to account for the fact that biomarker changes are influenced by the treatment and post-randomization factors. A key statistical challenge is that we do not know whether the observed biomarker change in an individual patient truly reflects a treatment-induced effect or whether the change would have occurred under placebo as well. This uncertainty makes it difficult to disentangle the causal effect of the treatment from natural biomarker variability. We apply principal stratification with a normal copula governed by the correlation ρ between the potential acute biomarker changes under treatment and placebo. A flexible model for the conditional distribution of the clinical endpoint given the biomarker change enables estimation of the conditional average treatment effect on the clinical endpoint, given the acute biomarker change under treatment, as a function of ρ. We illustrate the method by determining how knowledge of acute change in estimated glomerular filtration rate modifies the expected effect of sodium-glucose cotransporter-2 inhibitors (SGLT-2i) on clinical endpoints in patients with chronic kidney disease.

## 1 Introduction

Randomized clinical trials (RCTs) often evaluate causal treatment effects by employing an intent-to-treat approach, which asks, “What is the average effect of treatment assignment across the study population?” In certain cases, treatments may cause potentially adverse changes in a prognostic biomarker for a clinically relevant endpoint shortly after treatment initiation. In these cases, clinicians may question whether they should change the treatment.

The motivation of this paper stems from recent randomized clinical trials which have demonstrated that treatment with sodium-glucose cotransporter-2 inhibitors (SGLT-2i) slows disease progression and delays the onset of kidney failure in patients with chronic kidney disease (CKD) [1–5]. These studies also reported that some participants experience an acute reduction in estimated glomerular filtration rate (eGFR), a measure of renal function, within approximately 1 month after initiating treatment with SGLT-2i [5–7]. Although this drug class has been shown to slow the average progression of kidney disease at the population level, there is concern that this benefit may be reduced or even reversed in patients with unusually large acute eGFR declines.

From a biological perspective, initial eGFR declines after initiating SGLT-2is and certain other classes of medications may result from mechanisms that slow CKD progression in the long-run. This decline is thought to be driven primarily by reductions in intraglomerular pressure, which occur due to hemodynamic changes such as afferent arteriole constriction and efferent arteriole dilation. These adaptations help preserve long-term kidney function by reducing hyperfiltration and mitigating glomerular damage, ultimately slowing CKD progression [8]. In addition, recent epidemiologic analyses suggest that in patients with type 2 diabetes, initial reductions in eGFR following the initiation of SGLT-2i are not associated with an increased risk of adverse events [9,10]. However, such analyses may be subject to bias. For instance, if patients with large eGFR declines are more likely to have their treatment discontinued, the observed relationship may underestimate any potential risks associated with acute eGFR declines. Hence, the implications of such epidemiologic associations for the causal question of whether larger than average acute eGFR declines are indicative of enhanced or reduced long term benefit are unclear. Our paper seeks insights into the implications of acute eGFR changes (ΔeGFR) under SGLT-2i treatment for the expected causal effect of SGLT-2i treatment on long-term clinical outcomes.

Empirical evaluation of the implications of ΔeGFR following treatment initiation is challenging. Progress in understanding how treatment effects are modified by post-treatment factors has been made through the framework of principal stratification. In this framework, causal effects are evaluated after conditioning on subsets of patients defined by counterfactual (potential) values of an intermediate variable that would have occurred under designated levels of the treatment [11]. Because principal strata are determined by counterfactuals, they can be viewed as defined at baseline, prior to the treatment, allowing average causal effects to be defined on those subgroups. In this case, our goal is to estimate the average causal effect of treatment with SGLT-2 inhibition within the principal strata defined by designated early changes in eGFR that would occur with the treatment. These principal strata are empirically determined only for patients assigned to the SLGT-2i intervention; they are not observed for patients assigned to the placebo. Thus, the challenge of using potential outcomes in principal stratification arises from their partial observability in a parallel-arm RCT. To estimate treatment effects within these principal strata, we must rely on additional identifying assumptions based on subject matter considerations that cannot be verified from the data. Often, a monotonicity assumption is applied in similar problems, usually in conjunction with a sensitivity analysis to evaluate how the average causal effect of interest depends on non-identified parameters. In our context, a common form of the monotonicity assumption would posit that for all patients, the decline in eGFR that would be observed with SGLT-2i treatment is at least as large as the decline in eGFR that would be observed under placebo. For complex diseases such as CKD, the assumption that the treatment modifies the eGFR change in the same direction for all patients is a stretch. Treatment with SGLT-2i is likely to decrease eGFR by some mechanisms but increase eGFR by others; while a greater mean eGFR decline is observed in the short term after initiating SGLT-2i, it is difficult to rule out the possibility that the treatment may lead to short-term eGFR increases, at least by small amounts, in a subset of patients. A recent cross-over study [12] illustrates the limitations of the conventional monotonicity assumption by showing its inapplicability when potential outcomes under both interventions can be observed in a controlled setting.

Most approaches proposed to evaluate the causal effect in the principal stratification framework have focused on binary or discrete post-treatment variables [13–20]. However, in real-world applications, biomarkers with acute responses to treatments, including eGFR in response to SGLT-2i, are often continuous. When dealing with continuous post-treatment variables, the number of potential principal strata becomes infinite, complicating both inference and interpretation. Several approaches have been proposed in the causal inference literature to address these challenges. One approach is to dichotomize the post-treatment variable [21,22], though this method discards information provided by the continuous scale of the biomarker and introduces issues with inference due to the arbitrary selection of thresholds. Another strategy assumes strong parametric models for both the outcome and the post-treatment variable and jointly modeling them with informative priors for the parameters [23,24]. A different line of development relaxes these parametric assumptions by using a Dirichlet process mixture model within a Bayesian framework [25]. Two recent developments extend nonparametric modeling in the Bayesian framework; one does so by incorporating Gaussian processes in the case of continuous treatment [26] and the other allows for information sharing across treatment groups in determining principal strata membership [27]. While these methods offer potential solutions for handling continuous post-treatment variables, they often require strong assumptions or produce estimands that are difficult to interpret.

An alternative line of research relaxes the restrictive monotonicity assumption by employing copula [28] functions to model the bivariate joint distribution of potential values for continuous post-treatment variables across the treatment and placebo interventions. This copula-based approach has been proposed for modeling principal strata across various contexts (e.g., [23,24,29–31]).

These copula-based approaches show promise but may produce unstable estimates when identifiability is not assured [24]. In this paper, we replace the assumption of strict monotonicity by positing a bivariate normal copula for the joint distribution of the counterfactual acute eGFR changes that would be observed with and without the treatment, while treating the correlation between these changes as a sensitivity parameter. We use the Johnson family of distributions [32] to flexibly model the distribution of ΔeGFR, and adjust for baseline covariates to address possible confounding. We apply a competing risks framework to characterize to the extent possible from the data, how the expected effect of SGLT-2i on the time to kidney failure (with death as competing event) is modified by knowledge of the acute eGFR change following SGLT-2i initiation, while also articulating the uncertainty resulting from the dependence of this conditional expectation on causal assumptions that extend beyond the observable data. The paper is organized as follows: Sect 2 provides a heuristic overview of key concepts. Sect 3 introduces our method, detailing the estimand of interest, the identifying causal assumptions, the analytical approach, and the estimation algorithm. Sect 4 provides additional details of our approach for the specific application to clinical trials of SGLT-2i in patients with chronic kidney disease. We provide the results in Sect 5, and Sect 6 is devoted to discussing these findings and conclusions.

## 2 Heuristic overview

**Fig 1:**
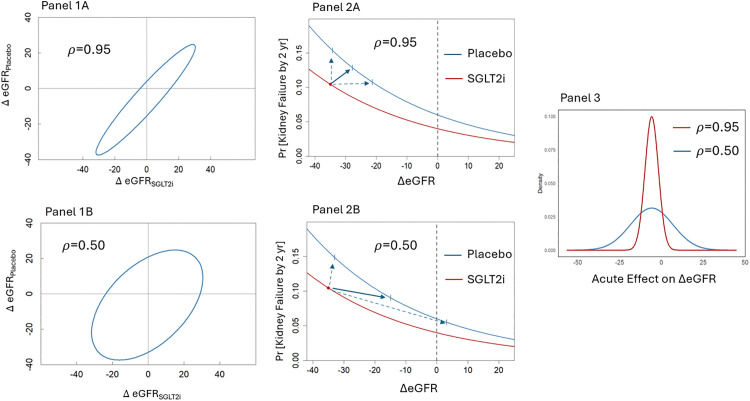
Heuristic Overview of the framework. The figure illustrates how the acute effect relates to the potential outcomes $\Delta {\text{eGFR}}_{\text{SGLT-2i}}$ and $\Delta {\text{eGFR}}_{\text{placebo}}$ for lower (ρ=0.5) and higher (ρ=0.95) values of ρ for a simplified scenario in which the potential outcomes follow a bivariate normal distribution. It also describes the implications of this relationship for the longer term treatment effect on a clinical endpoint. Panel 1 compares the joint distribution between $\Delta {\text{eGFR}}_{\text{SGLT-2i}}$ and $\Delta {\text{eGFR}}_{\text{placebo}}$ between the two values of ρ. Panel 2 shows how ρ influences the conditional average treatment effect on the clinical endpoint given an observed acute eGFR decline of 35%, and Panel 3 demonstrates the relationship between ρ and the variability of the acute effect.

Before beginning our formal development, we first give a heuristic overview of the framework. Panels 1A and 1B of Fig 1 illustrate two possible models for the association between the percent change in eGFR on placebo ($\Delta {\text{eGFR}}_{\text{placebo}}$) and the percent change in eGFR on SGLT-2i ($\Delta {\text{eGFR}}_{\text{SGLT-2i}}$). In both panels, we set the mean percent changes under placebo and SGLT-2i to –0.6% and –6.4%, respectively, and the standard deviation in both arms to 12.7%, to correspond roughly to the data presented later in the manuscript. However, the correlation ρ between $\Delta {\text{eGFR}}_{\text{placebo}}$ and $\Delta {\text{eGFR}}_{\text{SGLT-2i}}$ cannot be determined empirically because the two quantities cannot be observed simultaneously. As a result, we cannot know whether the potential outcomes are tightly correlated, as in Panel 1A, or weakly correlated, as in Panel 1B.

Next, consider a scenario in which a 35% eGFR reduction is observed one month after initiating SGLT-2i therapy, and in which SGLT-2i reduces the 2-year risk of kidney failure by one-third at every value of the observed ΔeGFR . Larger declines are assumed to be associated with higher risk of kidney failure, as in Panels 2A and 2B. Under a strong correlation (ρ=0.95), the expected value of $\Delta {\text{eGFR}}_{\text{placebo}}$ corresponding to a 35% decline on SGLT-2i is only slightly attenuated to –27.8%, and the distribution of possible $\Delta {\text{eGFR}}_{\text{placebo}}$ values is relatively narrow (Panel 2A). In contrast, under a weaker correlation (ρ=0.50), the expected $\Delta {\text{eGFR}}_{\text{placebo}}$ shifts more substantially to –14.9%, and the distribution becomes much wider (Panel 2B). Below we show that, under a no-confounding assumption, and for any specified ρ, the average treatment effect conditional on an observed 35% eGFR decline after starting SGLT-2i can be estimated by comparing (i) the 2-year probability of kidney failure with SGLT-2i to (ii) the *average* 2-year probability of kidney failure with placebo across the conditional distribution of $\Delta {\text{eGFR}}_{\text{placebo}}$ given the observed $\Delta {\text{eGFR}}_{\text{SGLT-2i}}$. This latter quantity corresponds approximately to the vertical coordinate of the blue curve in Panels 2A or 2B at –27.8% (Panel 2A) or –14.9% (Panel 2B). In the illustrated scenario, an observed 35% decline is associated with a beneficial expected treatment effect when the potential outcomes are strongly correlated (Panel 2A), but with a harmful expected effect when they are weakly correlated (Panel 2B). Thus, the data alone cannot determine whether such a decline indicates benefit or harm; the answer depends on ρ, which is not identifiable from the data.

However, subject-matter knowledge may help constrain plausible values of ρ. Lower correlations imply larger variability in the acute SGLT-2i effect given by the difference $\Delta {\text{eGFR}}_{\text{SGLT-2i}}-\Delta {\text{eGFR}}_{\text{placebo}}$, and therefore imply that substantial fractions of patients have large *positive* acute effects (Panel 3). If physiological considerations preclude substantial positive acute effects in a large share of patients, this would imply a lower bound to ρ, potentially ruling out the harmful-effect scenario.

We next describe our formal framework, which extends this heuristic overview by allowing non-normal ΔeGFR  and incorporating covariate adjustment to account for possible confounding.

## 3 Methods

### 3.1 Notations and estimand of interest

Consider a randomized trial where participants are randomized to SGLT-2i treatment or placebo. For the purposes of this manuscript, our primary objective is to understand the implications of acute eGFR change for the effect of the treatment on the occurrence of kidney failure and all-cause mortality, where kidney failure and all-cause mortality are competing events. For the subsequent development, unless stated, we refer to kidney failure (with all-cause mortality prior to kidney failure considered as a competing risk) as the primary clinical endpoint. This reflects the central clinical concern that acute effects on eGFR may hasten progression of kidney disease. Acute eGFR changes may also affect deaths from cardiovascular disease and other causes, but the mechanisms are less clear. Hence, all-cause mortality and the composite of kidney failure and all-cause mortality will be the secondary outcomes.

To formalize this framework statistically, let *T* and *C* respectively denote the failure and censoring times for the composite of kidney failure or death, and let E∈(1,2) indicate whether the observed event is kidney failure (*E* = 1) or all-cause mortality (*E* = 2). We define acute eGFR change, ΔeGFR, as the percentage change in eGFR from baseline to month 1, using the 2009 CKD-EPI equation [33]. For each subject *i*, the observed data consist of $Y_{i}=\min\left(T_{i},C_{i}\right)$, the time to either an event or censoring; $\delta_{i}=I\left(T_{i}\leq C_{i}\right)$, a binary indicator of whether the event was observed; *A*_*i*_, the randomized treatment assignment (1 for SGLT-2i and 0 for placebo); and **X**_**i**_, a vector of baseline covariates. In a competing risks framework, we additionally observe *E*_*i*_ for those who have either events, such that the full data structure is ($Y_{i},\delta_{i},E_{i},𝐗$, *A*_*i*_). When the interest lies in modeling the cumulative incidence function for kidney failure conditional on covariates, the inference centers on $F_{1}(t;𝐗)=\mathrm{Pr}(T\leq t,E=1|𝐗)$.

To address causal effects using potential outcomes, let *T*_*i*_(1) and *T*_*i*_(0) denote the time to the clinical endpoint for the *i^th^* subject under SGLT-2i and placebo, respectively. Similarly, let *W*_*i*_(1) and *W*_*i*_(0) denote the ΔeGFR for the *i^th^* subject under SGLT-2i and placebo. Note that we observe *W*_*i*_(1) and *T*_*i*_(1) only for patients randomized to the treatment, and we observe *W*_*i*_(0) and *T*_*i*_(0) only for patients randomized to the placebo. It is important to distinguish between *W*_*i*_(1), which represents the acute change in eGFR after initiating SGLT-2i, and *W*_*i*_(1) − *W*_*i*_(0), which represents the acute effect of SGLT-2 inhibition. We are able to observe *W*_*i*_(1) in patients assigned to SGLT-2i and *W*_*i*_(0) in placebo patients, but we cannot observe the acute effect *W*_*i*_(1) − *W*_*i*_(0) in the same patient. Also let *E*_*i*_(1) and *E*_*i*_(0) be potential event type indicators for the *i^th^* subject under SGLT-2i and placebo respectively.

We express the treatment effect estimand for the composite outcome of kidney failure and all-cause mortality as a difference in cumulative risks at time *k*:


$$\Delta \left(k|w_{1}\right)=\mathrm{Pr}\left[T(1)\leq k|W(1)=w_{1}\right]-\mathrm{Pr}\left[T(0)\leq k|W(1)=w_{1}\right]$$ (1)

For the competing outcomes (kidney failure and all-cause mortality), the estimand is:


$$\Delta_{e}\left(k|w_{1}\right)=\mathrm{Pr}\left[T(1)\leq k,E(1)=e|W(1)=w_{1}\right]-\mathrm{Pr}\left[T(0)\leq k,E(0)=e|W(1)=w_{1}\right].$$ (2)

Because a patient must survive without kidney failure for 1 month for the acute eGFR change to be observed, more precise expressions for the estimands in (1) and (2) must also condition on survival without kidney failure to 1 month. Thus, a more formal expression for the second estimand is


$$\begin{array}{l} \Delta_{e}\left(k|w_{1}\right)=\\ \;\mathrm{Pr}\left[T(1)\leq k,E(1)=e|W(1)=w_{1},T(1)\geq 1\text{ month }\right]-\\ \;\mathrm{Pr}\left[T(0)\leq k,E(0)=e|W(1)=w_{1},T(1)\geq 1\text{ month }\right] \end{array}$$

As only 0.02% of patients experienced events within the first month, for simplicity we subsequently omit the formal conditioning on patients surviving without kidney failure from our notation.

### 3.2 Analytic approach and causal assumptions

Focusing on the estimand in expression (2) above, the first term, $\mathrm{Pr}\left[T(1)\leq k,E(1)=e|W(1)=w_{1}\right]$, can be estimated directly from the data of patients randomized to SGLT-2i, but the comparator $\mathrm{Pr}\left[T(0)\leq k,E(0)=e|W(1)=w_{1}\right]$ cannot be identified solely from the observed data since we never observe *W*_*i*_(1) and *T*_*i*_(0) in the same patient. Thus, to make progress, we make causal assumptions from subject matter considerations that go beyond our actual data. To do this, we employ a bivariate normal copula to characterize the joint distribution of the counterfactual ΔeGFR values, *W*_*i*_(0) and *W*_*i*_(1).

#### 3.2.1 Transformation with Normal copula.

Let *F*_(0)_ and *F*_(1)_ denote the cumulative distribution functions for *Wi*(0) and *Wi*(1), respectively, and let $F_{\left(0\right)}^{-1}$ and $F_{\left(1\right)}^{-1}$ denote their inverses. We use the Johnson family of distributions to model *F*_(0)_ and *F*_(1)_; this family includes the normal distribution as a special case but also allows for non-zero skewness and kurtosis. Let Φ denote the cumulative distribution function for the standard normal distribution with mean 0 and standard deviation 1. Define $Y_{i}(0)=\Phi^{-1}\left[F_{\left(0\right)}\left(W_{i}(0)\right)\right]$ and $Y_{i}(1)=\Phi^{-1}\left[F_{\left(1\right)}\left(W_{i}(1)\right)\right]$. Then *Y*_*i*_(0) and *Y*_*i*_(1) each have standard normal distributions. Further, since Φ has a known form, we can directly estimate both *Y*_*i*_(0) and *Y*_*i*_(1) from the data.

Identification of $\mathrm{Pr}\left[T(0)\leq k,E(0)=e|W(1)=w_{1}\right]$ in expression 2 requires additional assumptions.

#### 3.2.2 Identifiability.

To obtain causal identification of $\Delta_{e}\left(k|w_{1}\right)$, we make the following assumptions.

**Assumption 1:** *Y*_*i*_(0) and *Y*_*i*_(1) follow a bivariate normal distribution.

**Assumption 2:** The correlation between *Y*_*i*_(0) and *Y*_*i*_(1) is equal to a value ρ which will serve as a sensitivity parameter.

**Assumption 3:**


$$\begin{array}{l} Pr\left[T_{i}(0)\leq k,E_{i}(0)=e_{i}|W_{i}(1)=w_{1},W_{i}(0)=w_{0}\right]\\ =Pr\left[T_{i}(0)\leq k,E_{i}(0)=e_{i}|W_{i}(0)=w_{0}\right] \end{array}$$

Assumption 3 essentially states that knowing the acute effect for a patient provides no additional information on the patient’s time to kidney failure or death in the absence of SGLT-2i after accounting for the patient’s ΔeGFR in the absence of SGLT-2i. In Sect 3.3, we show that the plausibility of this assumption can be strengthened by incorporating adjustment for potential confounders that could jointly influence the acute effect and CKD progression under the placebo. From Assumptions 1 and 2, it follows that the conditional distribution of *Y*_*i*_(0) given *Y*_*i*_(1) = *y* is $N\left(\rho y,\sqrt{1-\rho^{2}}\right)$. Hence


$$F_{W_{i}(0)|W_{i}(1)}(w_{0}|w_{1})=\Phi \left(\frac{\Phi^{-1}[F_{\left(0\right)}(w_{0})]-\rho \Phi^{-1}[F_{\left(1\right)}(w_{1})]}{\sqrt{1-\rho^{2}}}\right).$$ (3)

In what follows, we concentrate on the causal identification of $\Delta_{e}\left(k|w_{1}\right)$. Specifically, using the assumptions above, we can modify the second term in 2 as;


$$\begin{array}{l} \mathrm{Pr}\left[T(0)\leq k,E(0)=e|W(1)=w_{1}\right]\\ =\int \mathrm{Pr}\left[T_{i}(0)\leq k,E_{i}(0)=e_{i}|W_{i}(1)=w_{1},W_{i}(0)=w_{0}\right]f\left(w_{0}|w_{1}\right)dw_{0}\\ =\int \mathrm{Pr}\left[T_{i}(0)\leq k,E_{i}(0)=e_{i}|W_{i}(0)=w_{0}\right]f\left(w_{0}|w_{1}\right)dw_{0} \end{array}$$

where the first equation follows by the law of total probability and the last equation follows by invoking Assumption 3. Here $f\left(w_{0}|w_{1}\right)$ is the conditional density of *W*(0) given *W*(1).

To implement the integration in the final expression above, note that


$$\int \mathrm{Pr}\left[T_{i}(0)\leq k,E_{i}(0)=e_{i}|W_{i}(0)=w_{0}\right]f\left(w_{0}|w_{1}\right)dw_{0}$$ (4)

is the average of the conditional probabilities $\mathrm{Pr}[T_{i}(0)\leq k,E_{i}(0)=e_{i}$ | *W*_*i*_(0) = *w*_0_] over the conditional distribution of *W*_*i*_(0) given that *W*_*i*_(1) = *w*_1_. Thus, we can approximate the integral in 4 as:


$$\begin{array}{l} \int \mathrm{Pr}\left[T_{i}(0)\leq k,E_{i}(0)=e_{i}|W_{i}(0)=w_{0}\right]f\left(w_{0}|w_{1}\right)dw_{0}\\ \approx \underset{j}{\underbrace{\text{ Ave }}}\left\{\mathrm{Pr}\left[T_{i}(0)\leq k,E_{i}(0)=e_{i}|W_{i}(0)=w_{0,i,j}\right]\right\} \end{array}$$

where the *w*_0,*i*, *j*_; *j* = 1,2, … *J* are simulated randomly from the conditional distribution of *W*_*i*_(0) given $W_{i}(1)=w_{1}$. We can make the approximation as accurate as desired by increasing the number of draws from the conditional distribution.

### 3.3 Covariate adjustment

As described above, Assumption 3 stipulates that a patient’s acute effect provides no additional information on the patient’s time to kidney failure or death without SGLT-2i after accounting for the patient’s ΔeGFR in the absence of SGLT-2i. This is a type of no-confounding assumption between the acute effect of the treatment and the long-term rate of CKD progression. Assumption 3 may be more plausible with adjustment for baseline covariates that subject matter considerations suggest may be related to both the acute effect and rate of CKD progression. We therefore modify Assumptions 1−3 to incorporate covariate adjustment.

Let *X*_*i*_ denote a collection of baseline covariates. Let $W_{i}(1)=X_{i}\beta (1)+r_{i}(1)$ and $W_{i}(0)=X_{i}\beta (0)+r_{i}(0)$, where $E\left(W_{i}(1)|X_{i}\right)=X_{i}\beta (1)$ and $E\left(W_{i}(0)|X_{i}\right)=X_{i}\beta (0)$, and *r*_*i*_(1) and *r*_*i*_(0) are the residuals in the two regressions. Let $\epsilon_{i}(0)=\Phi^{-1}\left[F_{r(0)}\left(r_{i}(0)\right)\right]$ and $\epsilon_{i}(1)=\Phi^{-1}\left[F_{r(1)}\left(r_{i}(1)\right)\right]$, where *F*_*r*(0)_ and *F*_*r*(1)_ are the cdf’s of *r*(0) and *r*(1), respectively. We can then replace Assumptions 1–3 with similar assumptions expressed in terms of the $\epsilon_{i}(0)$ and $\epsilon_{i}(1)$.

**Assumption 1B**: $\epsilon_{i}(0)$ and $\epsilon_{i}(1)$ follow a bivariate normal distribution.

**Assumption 2B**: The correlation between $\epsilon_{i}(0)$ and $\epsilon_{i}(1)$ is equal to a value $\rho_{\epsilon }$ that we treat as a sensitivity parameter.

Note that the difference between **assumptions 1B and 2B** vs. **1 and 2** is that the $\epsilon_{i}$’s are the transformed residuals from regressing each of *W*_*i*_(0) and *W*_*i*_(1) on the covariates in the treatment groups.

**Assumption 3B:**


$$\begin{array}{l} Pr\left[T_{i}(0)\leq k,E_{i}(0)=e_{i}|W_{i}(1)=w_{1},W_{i}(0)=w_{0},X_{i}=x_{i}\right]\\ \;=Pr\left[T_{i}(0)\leq k,E_{i}(0)=e_{i}|W_{i}(0)=w_{0},X_{i}=x_{i}\right]. \end{array}$$

By the law of total probability, the estimands 1 and 2 can be expressed:


$$\Delta^{*}\left(k|w_{1}\right)=\int \left\{\mathrm{Pr}\left[T_{i}(1)\leq k|W_{i}(1)=w_{1},X_{i}=x_{i}\right]f\left(X_{i}=x_{i}|w_{1}\right)\right\}dx\;-\int \left\{\mathrm{Pr}\left[T_{i}(0)\leq k|W_{i}(1)=w_{1},X_{i}=x_{i}\right]f\left(X_{i}=x_{i}|w_{1}\right)\right\}dx$$ (5)

$$\Delta_{e}^{*}\left(k|w_{1}\right)=\int \left\{\mathrm{Pr}\left[T_{i}(1)\leq k,E_{i}(1)=e_{i}|W_{i}(1)=w_{1},X_{i}=x_{i}\right]f\left(X_{i}=x_{i}|w_{1}\right)\right\}dx\;-\int \left\{\mathrm{Pr}\left[T_{i}(0)\leq k,E_{i}(0)=e_{i}|W_{i}(1)=w_{1},X_{i}=x_{i}\right]f\left(X_{i}=x_{i}|w_{1}\right)\right\}dx$$ (6)

The estimand in 6 can be identified by another application of the law of total probability and invoking assumption 3B as follows:


$$\begin{array}{l} \Delta_{e}^{*}\left(k|w_{1}\right)\\ \;=\int \{\mathrm{Pr}\left[T_{i}(1)\leq k,E_{i}(1)=e_{i}|W_{i}(1)=w_{1},X_{i}=x_{i}\right]f\left(X_{i}=x_{i}|w_{1}\right)\}dx\\ \;\;-\iint \{\mathrm{Pr}\left[T_{i}(0)\leq k,E_{i}(0)=e_{i}|W_{i}(1)=w_{1},W_{i}(0)=w_{0},X_{i}=x_{i}\right]f\left(w_{0}|w_{1},x\right)\\ \;\;\;f\left(X_{i}=x_{i}|w_{1}\right)dw_{0}dx\}\\ \;=\int \{\mathrm{Pr}\left[T_{i}(1)\leq k,E_{i}(1)=e_{i}|W_{i}(1)=w_{1},X_{i}=x_{i}\right]f\left(X_{i}=x_{i}|w_{1}\right)\}dx\\ \;\;-\iint \{\mathrm{Pr}\left[T_{i}(0)\leq k,E_{i}(0)=e_{i}|W_{i}(0)=w_{0},X_{i}=x_{i}\right]f\left(w_{0}|w_{1},x\right)\\ \;\;\;f\left(X_{i}=x_{i}|w_{1}\right)dw_{0}dx\} \end{array}$$

where $f\left(w_{0}|w_{1},x\right)$ is the conditional probability density function that defines the conditional distribution of *W*_*i*_(0) given *W*_*i*_(1) and *X*_*i*_ = *x*_*i*_. The is equivalent to the distribution of the sum of $x_{i}\beta (0)$ and a random variable whose distribution follows the conditional distribution of *r*_*i*_(0) given *r*_*i*_(1) and *x*_*i*_.

The quantity $f\left(x|w_{1}\right)$ represents the conditional probability density function that defines the conditional distribution of *X*_*i*_ given *W*_*i*_(1). To approximate this conditional distribution, we will employ the Sampling-Importance-Resampling (SIR) algorithm [34]. This approach provides a more robust and flexible framework for approximating $f\left(x|w_{1}\right)$ than attempting to fit a multivariate regression of *X* on *w*_1_ which would have to account for disparate types of variables (e.g., continuous and categorical) as well as the relationships among these variables. Using Baye’s theorem, we express the conditional density as $f\left(x|w_{1}\right)=\frac{f\left(w_{1}|x\right)f(x)}{f\left(w_{1}\right)}$, where $f\left(w_{1}|x\right)$ is the conditional pdf of *w*_1_ given *X* and *f*(*x*) is the unconditional pdf of the covariates *X*. The denominator $f\left(w_{1}\right)$ serves at the normalizing constant and is obtained by integrating the joint density over all possible values of *X*. To implement the SIR algorithm, we first generate proposal samples *x* from a multivariate normal distribution stratified by any dichotomous covariates. Each sample will then be assigned an importance weight based on the likelihood of observing *W*_1_ = *w*_1_, thereby adjusting for differences between the proposal and target distributions. The weights will be computed as $f\left(w_{1}|x\right)$ (approximated via regression of *w*_1_ on *x*) and the unconditional pdf *f*(*x*) (estimated from the observed data), with normalization ensuring they sum to one. Finally, we will perform resampling from the proposal distribution using these importance weights, effectively generating a new set of samples that more accurately reflects $f\left(x|w_{1}\right)$.

We are now able to define Algorithm 1, which provides the analytical steps needed to estimate the quantities in 6.

**Algorithm 1: Estimate $\Delta_{e}(k|w_{1})$ with covariate adjustment**

**Step 1:** Fit regression models $W_{i}(1)=X_{i}\beta (1)+r_{i}(1)$ and $W_{i}(0)=X_{i}\beta (0)+r_{i}(0)$, recording estimates of the coefficients β(1),β(0) and residuals *r*_*i*_(1), *r*_*i*_(0). Define a grid of *w*_1_ values.

**Step 2:** Fit a Fine-Gray regression [35] (for competing outcomes) or Cox regression (non-competing outcomes) relating *T*_*i*_(1) jointly to *W*_*i*_(1) and *X*_*i*_.

2a: Do the following for each *w*_1_ value in the grid of *w*_1_ values:

2ai:  Using the SIR algorithm, draw ${\hat{x}}_{i}$ from $f\left(x|w_{1}\right)$

2aii:  Apply the Breslow method [36] to the model in **Step 2** to provide estimates


$$\hat{Pr}\left[T_{i}(1)\leq k,E_{i}(1)=e_{i}|W_{i}(1)=w_{1},X_{i}={\hat{x}}_{i}\right]$$

**Step 3:** Fit a Fine-Gray (or Cox) regression relating *T*_*i*_(0) jointly to *W*_*i*_(0) and *X*_*i*_.

3a: Do the following for each *w*_1_ value in the grid of *w*_1_ values:

3ai:  Compute ${\hat{r}}_{1i}=w_{1}-{\hat{x}}_{i}\hat{\beta }(1)$.

3aii:  Compute ${\hat{\epsilon }}_{1i}=\Phi^{-1}\left({\hat{F}}_{r(1)}\left({\hat{r}}_{1i}\right)\right)$, where ${\hat{F}}_{r(1)}$ is estimated using the residuals $\left(r_{i}(1)\right)$ from the regression, $W_{i}(1)=X_{i}\beta (1)+r_{i}(1)$ in the SGLT- 2 i group.

3aiii:  Simulate a random sample of *J* values of ${\hat{\epsilon }}_{0ij},j=1,2,\ldots J$ for each *i* = 1,2, … *n* from the Normal distribution; ${\hat{\epsilon }}_{0ij}\sim N\left(\rho_{\epsilon }{\hat{\epsilon }}_{1i},\sqrt{1-\rho_{\epsilon }^{2}}\right)$.

3aiv:  Compute ${\hat{r}}_{0ij}={\hat{F}}_{r(0)}^{-1}\left[\Phi \left({\hat{\epsilon }}_{0ij}\right)\right]$, where ${\hat{F}}_{r(0)}$ is estimated using the residuals $\left(r_{i}(0)\right)$ from the regression, $W_{i}(0)=X_{i}\beta (0)+r_{i}(0)$ in the placebo group.

3av:  Compute $w_{0ij}={\hat{x}}_{i}\hat{\beta }(0)+{\hat{r}}_{0ij}$.

3avi:  Apply the Breslow method to the model in Step 3 to provide estimates $\hat{\mathrm{Pr}}\left[T_{i}(0)\leq k,E_{i}=e_{i}|W_{i}(0)=w_{0ij},X_{i}={\hat{x}}_{i}\right]$.

3b: Estimate $\Delta_{e}(k|w_{1})$ as the difference:


$$\begin{array}{l} \;\underset{i}{Ave}\left\{\hat{P}r\left[T_{i}(1)\leq k,E_{i}(1)=e_{i}|W_{i}(1)=w_{1},X_{i}={\hat{x}}_{i}\right]\right\}\\ -\underset{ij}{Ave}\left\{\hat{P}r\left[T_{i}(0)\leq k,E_{i}(0)=e_{i}|W_{i}(0)=w_{0ij},X_{i}={\hat{x}}_{i}\right]\right\} \end{array}$$

**Step 4:** Bootstrap the whole process to estimate pointwise 95% CIs for $\Delta_{e}(k|w_{1})$

### 3.4 Bound on $\rho_{\epsilon }$

To evaluate plausible bounds on $\rho_{\epsilon }$, we used a simulation-based procedure to reconstruct the joint distribution of the acute eGFR changes under placebo and SGLT-2i while preserving the observed marginal distributions within each treatment arm.

We first performed separate regressions of the acute percent changes in eGFR on baseline eGFR, log-proteinuria, age, systolic blood pressure, sex, and diabetes status in the SGLT-2i and placebo groups. This yielded arm-specific conditional mean functions $E(W(a)|X)=X\beta_{a}$, where *W*(*a*) denotes the acute change under treatment a∈{0,1}. Residuals were extracted from each model to represent unexplained variation in acute change after adjustment for measured covariates. We then fit Johnson SU distributions separately to the residuals in the placebo and SGLT-2i arms to map the residual distributions to standard normal distributions.

To construct a joint distribution for (*W*(0), *W*(1)) under a candidate value of $\rho_{\epsilon }$, we simulated pairs of standardized residuals from a bivariate normal distribution with mean zero, unit variances, and correlation $\rho_{\epsilon }$. These latent normal draws were then transformed to the original residual scale using the inverse Johnson SU cumulative distribution functions for each arm, preserving the empirically estimated marginal residual distributions while imposing the specified cross-arm dependence structure.

We constructed potential acute changes by adding the simulated residuals to the predicted mean acute change evaluated at representative covariate values (continuous covariates fixed at their sample means, with sex and diabetes indicators fixed at prespecified values). This yielded simulated draws of (*W*(0), *W*(1)) consistent with both the observed regression structure and the hypothesized residual correlation $\rho_{\epsilon }$.

For each value of $\rho_{\epsilon }$, we generated 15,000 simulated joint draws and computed the distribution of the individual acute effect ΔW=W(1)−W(0). We then evaluated the proportion of simulated patients whose acute effect exceeded a prespecified threshold (scaled to percentage change relative to mean baseline eGFR). By examining how this probability varied across values of $\rho_{\epsilon }$, we sought to determine the smallest correlation that is biologically plausible.

## 4 Data analysis

We included participants from three landmark SGLT-2i trials pivotal in demonstrating the benefits of SGLT-2i in slowing kidney disease progression.

The DAPA-CKD trial enrolled 4,304 CKD patients, with and without diabetes, randomized to dapagliflozin or placebo [3]. Dapagliflozin significantly reduced the risk of the primary outcome – a composite of 50% eGFR decline, kidney failure, death due to kidney failure, or cardiovascular mortality. Benefits were seen in both diabetic and nondiabetic groups, with similar adverse event rates in both treatment arms.

The CREDENCE trial involved 4,401 patients with type 2 diabetes and CKD, comparing canagliflozin to placebo [37]. Canagliflozin significantly lowered the risk of a primary composite outcome of 57% eGFR decline, kidney failure, death due to kidney failure, or cardiovascular mortality.

The EMPA-REG OUTCOME trial assessed 7,020 type 2 diabetes patients with cardiovascular disease, evaluating empagliflozin against placebo for three major adverse cardiovascular events (cardiovascular death, nonfatal myocardial infarction, or nonfatal stroke) and kidney disease progression [5]. Empagliflozin significantly reduced the risk of an exploratory endpoint of kidney failure and 57% eGFR decline.

In our analysis, we focused on three clinical endpoints: (1) kidney failure (with all-cause mortality as a competing event), (2) all-cause mortality (with kidney failure as a competing event), and (3) a composite of kidney failure and all-cause mortality. We defined ΔeGFR as the percentage change in eGFR from baseline to the measurement taken at the first visit. We pooled individual-level data from the three trials for analysis. Trial participants were then classified as receiving SGLT-2i vs. placebo based on the individual trial protocol definitions.

We estimate the estimand of interest (treatment effect, at *k* = 2 years) by implementing the methods outlined in Algorithm 1 in each of the 3 studies and overall. We confirmed the suitability of the proportional hazards assumption using the modified weighted Schoenfeld residuals [38]. For kidney failure and all-cause mortality outcomes, we used the Fine-Gray model to relate each clinical outcome to the ΔeGFR (with subdistribution hazards as a function of ΔeGFR modeled as restricted cubic splines with 4 knots). We used a similar Cox proportional hazard model to relate the composite outcome to ΔeGFR. We included baseline eGFR and log baseline proteinuria, which are markers of severity and aggressiveness of chronic kidney disease, as covariates. Based on subject matter considerations, we also adjusted for baseline SBP, age, sex and diabetes status (in the DAPA-CKD analysis). We estimated the treatment effect in the three trials separately, and for each clinical outcome, we pooled the effect estimates across the 3 studies using a weighted (by relative sample size) average of the trials’ effect estimates. We report the overall estimated treatment effects (and their 95% confidence intervals from 1000 bootstrap estimates) for all 3 clinical outcomes when ΔeGFR is 0% and when there is a decline of 20% for ρ values, 0.4,0.6,0.8, and 1.

We visualized the 2-year effect estimates for the kidney failure outcome analysis for ρ values ranging from 0 to 1 and varied across a broad range of values for ΔeGFR (−20% to 20%). We provide the same analyses for all-cause mortality and the composite outcome in the supplementary material.

## 5 Results

Across the three included landmark trials, EMPA-REG OUTCOME, DAPA-CKD, and CREDENCE, a total of 15,725 participants were initially enrolled. Of these, 470 participants were excluded: 349 due to missing key baseline variables (including creatinine, albumin-to-creatinine ratio [ACR], age, or sex) or privacy concerns, and 121 who lacked a post-randomization eGFR measurement within the first month. This resulted in a combined cohort of 15,255 individuals. To address extreme outliers in the distribution of acute eGFR changes, the cohort was further trimmed by the 0.5^*th*^ and 99.5^*th*^ quantiles, leaving a final analysis cohort of 15,101 participants (CREDENCE: 4,273; DAPA-CKD: 3,959; and EMPA-REG OUTCOME: 6,869).

The characteristics of participants by study and overall included in the analysis are shown in Table 1. Overall, the mean baseline eGFR was 61.9 ± 22.3ml/min/1.73 m^2^, with participants from EMPA-REG having the highest baseline eGFR on average (76.4 ± 19.8ml/min/1.73 m^2^). Across studies, EMPA-REG participants had the lowest median baseline proteinuria (17.7mg/g vs. DAPA CKD: 900.0mg/g, CREDENCE: 928.0 mg/g). Overall, the mean eGFR change from baseline was −3.9 ± 12.7% and ranged from 42.7% to 54.5%. CREDENCE recorded the highest percentage (6.4%) of kidney failure events, and EMPA-REG recorded the lowest percentage (0.4%). Overall, there were 954(6.0%) deaths and 537(4.0%) kidney failure events.

**Table 1:** Table of Characteristics by Study and Overall.

Characteristic	All	CREDENCE	EMPA-REG	DAPA-CKD
N	15101	4273	6869	3959
**Age (years):** mean (SD)	62.8 (9.8)	63.0 (8.6)	63.1 (8.6)	62.2 (12.0)
**Female sex:** n (%)	4713 (31.0%)	1438 (33.7%)	1952 (28.4%)	1323 (33.4%)
**Systolic BP (mmHg):** mean (SD)	137.2 (16.8)	140.1 (15.6)	135.4 (16.9)	137.1 (17.3)
**eGFR (ml/min/1.73m**^**2**^**):** mean (SD)	61.9 (22.3)	56.0 (16.9)	76.4 (19.8)	43.3 (12.4)
**Proteinuria (mg/g):** median (IQR)	373.9 (22.1, 1107.0)	928.0 (465.0, 1829.0)	17.7 (6.2, 71.6)	900.0 (500.0, 1900.0)
**Diabetes status:** n (%)	13857 (92%)	4273(100%)	6869(100%)	2715(68.6%)
**% eGFR change:** mean (SD)	−3.9(12.7)	−4.0(14.0)	−2.8(11.4)	−5.8(13.0)
**Kidney failure events:** n (%)	537 (4.0%)	274(6.4%)	26(0.4%)	237(6.0%)
**Death events:** n (%)	954 (6.0%)	352(8.2%)	393(5.7%)	209(5.3%)

eGFR: estimated glomerular filtration rate.

**Fig 2:**
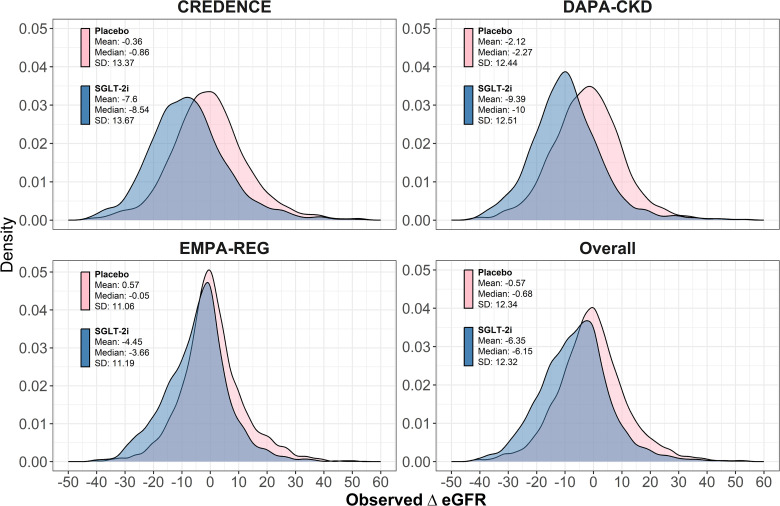
Kernel density of ΔeGFR by trial and overall. The figure illustrates that SGLT-2i treatment induces a significant acute decline in eGFR compared to placebo across all trials, which aligns with the known hemodynamic effects of these medications.

Combining data from all trials, the placebo group shows minimal acute change in eGFR (mean: −0.57%), while the SGLT-2i group has a mean decline of −6.35% (Fig 2). This consistent pattern across the trials indicates that SGLT-2i treatment is associated with a substantial acute decline in eGFR, while placebo generally shows smaller or even positive eGFR changes.

**Fig 3:**
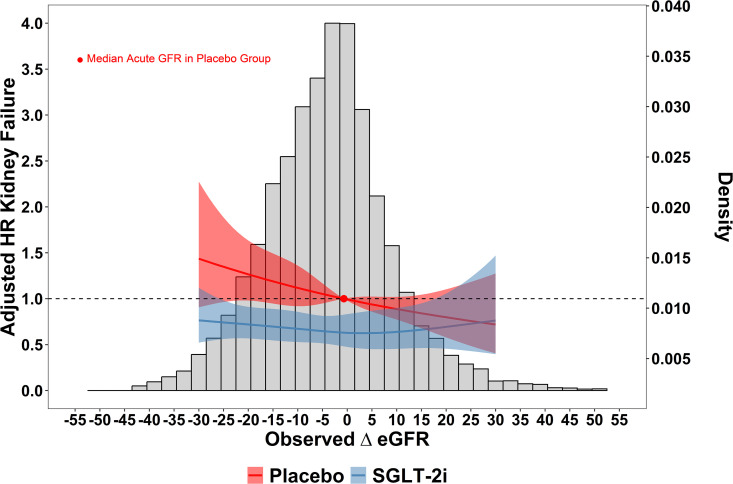
Empirical relationship between ΔeGFR and Kidney Failure. This shows the adjusted relationship between ΔeGFR and the sub-distribution hazard of kidney failure pooling across the three studies. The effect of ΔeGFR was modeled using a restricted cubic spline with 4 knots placed at the default locations of the rcs function in the rms package in R. We allowed for an interaction term between the restricted cubic splines of ΔeGFR at 1 month and treatment variable. We set the median ΔeGFR in the placebo group as the referent point. The models were adjusted for baseline eGFR, proteinuria, SBP, age, sex, and diabetes status. The shaded ribbons represent the 95% CI. The left y-axis provides the adjusted sub-distribution hazard ratio for every value of ΔeGFR compared to the reference point defined as the median ΔeGFR in the placebo group. The histogram of ΔeGFR is shown with the density on the right y-axis.

Fig 3 displays the cubic spline regressions relating the sub-distribution hazard for kidney failure to ΔeGFR by randomized group with adjustment for the covariates, using the median ΔeGFR in the placebo group as the reference. The hazard ratios were consistently less than 1 across the full ΔeGFR range in the SGLT-2i group.

**Fig 4:**
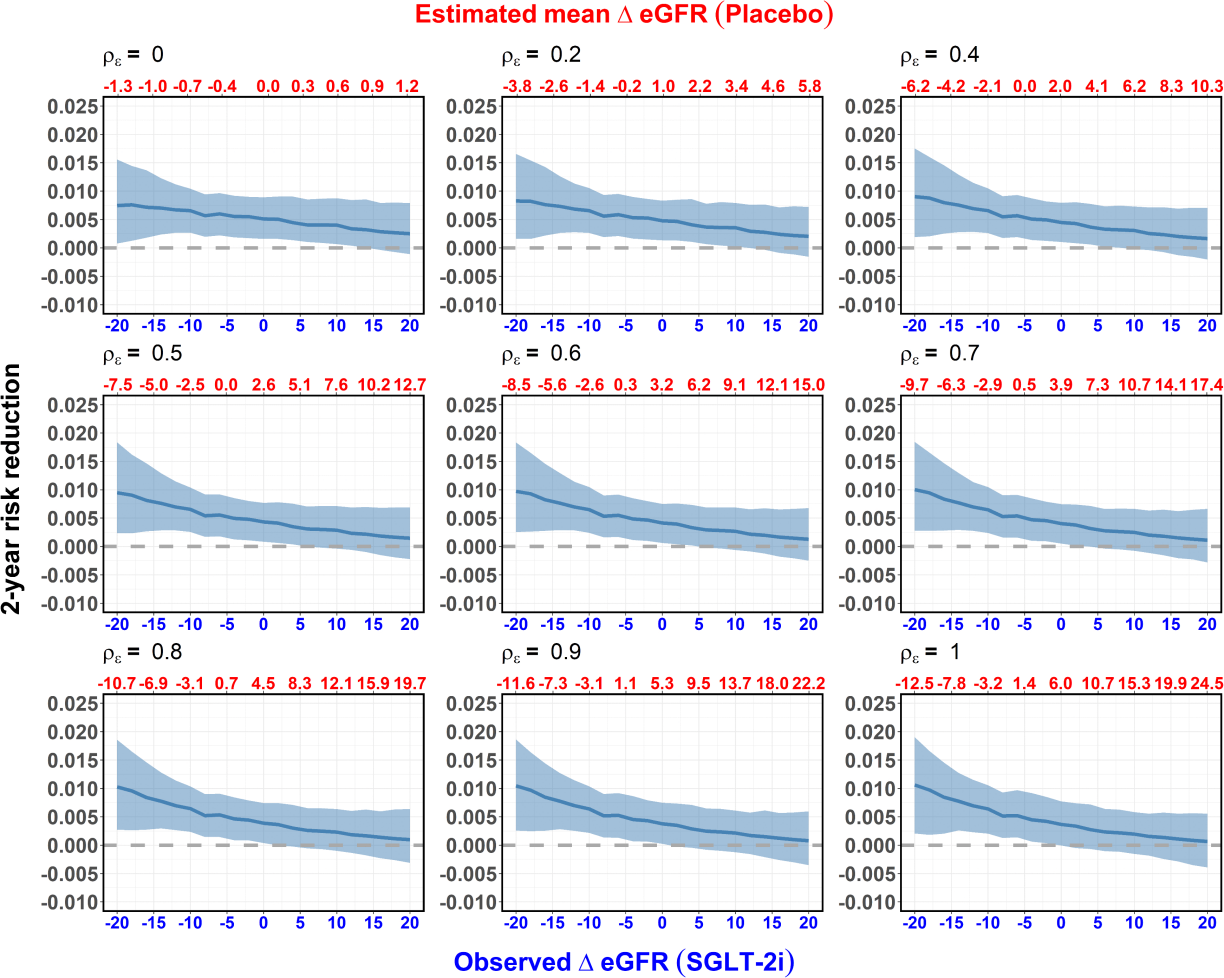
Implications of ΔeGFR on the estimated effect of SGLT-2i on the risk of kidney failure by 2 years. The figure displays the implications of the observed acute eGFR change (ΔeGFR) after starting SGLT-2i on the estimated effect of SGLT-2i on the risk of kidney failure within two years for alternative $\rho_{\epsilon }$. The analysis adjusts for baseline eGFR, log-transformed proteinuria, age, systolic blood pressure (SBP), sex and diabetes status. The relationship between ΔeGFR and kidney failure (with all-cause mortality as a competing event) was modeled using a restricted cubic spline with four knots. **The y-axis** shows the 2-year reduction in risk of kidney failure for patients treated with SGLT-2i compared to those without, using the steps outlined in Algorithm 1. **The bottom axis** represents the observed ΔeGFR after starting SGLT-2i, expressed as % change from baseline. **The top axis** indicates the corresponding estimated mean ΔeGFR with the placebo under the assumed value of $\rho_{\epsilon }$. **The steel blue line** shows the estimated reduction in the risk of kidney failure under the assumed $\rho_{\epsilon }$ given the observed ΔeGFR following SGLT-2i initiation, with 95% pointwise confidence intervals.

Fig 4 shows the estimated reduction in the risk of kidney failure by 2 years between patients with vs. without SGLT-2i given the observed ΔeGFR after starting SGLT-2i using the approach defined in Algorithm 1. In contrast to Fig 3, which depicts the directly observed relationship between the sub-distribution hazard for kidney failure and ΔeGFR, the estimated treatment effects in Fig 4 depend on the sensitivity parameter $\rho_{\epsilon }$, which cannot be determined from the data. However, across all values of $\rho_{\epsilon }$, we find no evidence that negative ΔeGFR after SGLT-2i initiation indicates either an adverse effect or a reduction in the benefit of SGLT-2i on kidney failure. Moreover, for larger values of $\rho_{\epsilon }$, we observe a trend suggesting that more negative acute changes signify a greater benefit of SGLT-2i on kidney failure. A similar pattern was observed for the composite of kidney failure and death, and an overall benefit of SGLT-2i on the competing risk of mortality free of kidney failure was seen irrespective of the the assumed value for $\rho_{\epsilon }$ (see Table 2, S2 Fig, and S4 Fig). To put the treatment effects displayed in Fig 4 into context, the absolute risk reduction of 0.010 at 2 -years corresponds to a relative risk reduction of approximately 30% compared to the 2 -risk risk of kidney failure in the control group of 0.034.

**Table 2:** Estimated Risk Reduction for Clinical Outcomes Given the Observed eGFR Decine After Starting SGLT-2i.

	20% Decline	No decline (0%)
**Kidney Failure**		
$\rho_{\epsilon }=0.4$	0.009 (0.002,0.018)	0.004 (0.001,0.008)
$\rho_{\epsilon }=0.6$	0.010 (0.003,0.018)	0.004 (0.001,0.007)
$\rho_{\epsilon }=0.8$	0.010 (0.003,0.019)	0.004 (0.000,0.007)
$\rho_{\epsilon }=1.0$	0.011 (0.002,0.019)	0.004(0.000,0.008)
**Death**		
$\rho_{\epsilon }=0.4$	0.014 (0.001,0.027)	0.014 (0.006,0.021)
$\rho_{\epsilon }=0.6$	0.015 (0.002,0.028)	0.013 (0.006,0.020)
$\rho_{\epsilon }=1.8$	0.014 (0.000,0.027)	0.012 (0.005,0.020)
$\rho_{\epsilon }=1.0$	0.013 (-0.002,0.026)	0.012 (0.004,0.021)
**Composite of Kidney Failure and Death**		
$\rho_{\epsilon }=0.4$	0.026 (0.011,0.041)	0.019 (0.010,0.029)
$\rho_{\epsilon }=0.6$	0.027 (0.011,0.043)	0.018 (0.009,0.027)
$\rho_{\epsilon }=1.8$	0.028 (0.011,0.044)	0.017 (0.008,0.027)
$\rho_{\epsilon }=1.0$	0.028 (0.010,0.044)	0.017 (0.007,0.027)

Shown are the estimated effects of SGLT-2i treatment on the probability of clinical outcomes by 2 years, given an observed eGFR decline of either 20% or 0% 1 month after initiating SGLT-2i. Results expressed as risk reductions.

We also formally addressed whether the conditional average treatment effects on the clinical endpoints at 2 years varied across different observed acute eGFR changes following SGLT-2i initiation. This was achieved by estimating the difference in the conditional average treatment effect at each ΔeGFR compared to the conditional average treatment effect when ΔeGFR=0 (See S1 Fig, S3 Fig, and S5 Fig). For kidney failure alone (S1 Fig) and for the composite of kidney failure and death (S5 Fig), larger acute declines in eGFR after starting SGLT-2i were associated with increased treatment benefit, with the trend becoming more pronounced as the sensitivity parameter $\rho_{\epsilon }$ increases (e.g., $\rho_{\epsilon }\geq 0.8$). However, the 95% confidence intervals for these differences largely overlap with zero across the spectrum of ΔeGFR, indicating that the trends for increased benefit for larger eGFR reductions did not reach statistical significance.

Table 3 shows the implications of the correlation parameter, $\rho_{\epsilon }$ for the variability in the acute effect of SGLT-2i and the associated predicted proportion of patients with an acute effect greater than $1ml/min/1.73{\;m}^{2}$. In this analysis, we converted the acute effect expressed as a percent to an absolute difference in eGFR levels to facilitate judgments as to the plausible size of the acute effect. As in the heuristic example displayed in Fig 1, smaller values for $\rho_{\epsilon }$ imply greater variation in the acute effect with a consequent increase in the proportion of patients with positive acute effects greater than $1\;ml/min/1.73{\;m}^{2}$.

**Table 3:** Estimated bounds on $\rho_{\epsilon }$.

$\rho_{\epsilon }$	Proportion of patients with acute effect $>1\;ml/min/1.73m^{2}$	Standard deviation of acute effect (%)
0.0	0.32	18.1
0.1	0.30	17.0
0.2	0.29	16.1
0.3	0.28	15.1
0.4	0.27	14.0
0.5	0.25	12.9
0.6	0.23	11.6
0.7	0.20	10.0
0.8	0.16	8.2
0.9	0.08	5.9
1.0	0.00	0.6

For values of $\rho_{\epsilon }\leq 0.8$, more than 16% of patients would exhibit an acute increase in eGFR greater than $1ml/min/1.73{\;m}^{2}$ within one month of treatment initiation. SGLT-2i’s could cause short-term increases in eGFR in some patients if their beneficial effect in slowing CKD progression produced a larger short term effect on eGFR change than their hemodynamic reduction in eGFR. However, eGFR reduction associated with CKD progression in the investigated populations is typically between 2 and 6 ml/min/1.73 m^2^ year, or less than 0.5 ml/min/1.73 m^2^ within 1 month. Thus, we are not aware of a plausible biological mechanism that would drive such a widespread improvement in kidney function within 1 month after starting SGLT-2i in a large proportion of patients. If widespread positive acute effects greater than $1\;ml/min/1.73{\;m}^{2}$ are considered unlikely, this analysis would suggest that $\rho_{\epsilon }$ is likely to be relatively high, probably ≥ 0.80.

## 6 Discussion

Nephrologists have often speculated that acute declines in eGFR following initiation of SGLT-2i and other medications that produce similar acute eGFR declines are benign and reversible [4,5,39]. Nonetheless, concerns about treatment safety and efficacy persist among clinicians and patients. This paper evaluates the implications of the observed acute eGFR change for the treatment’s longer-term effects. Recognizing that the true acute effect is unobserved, we use the framework of principal stratification to make explicit the inherent limits in what the data reveal. We characterize the key uncertainty in our understanding through the sensitivity parameter, $\rho_{\epsilon }$, which defines the correlation between suitably transformed potential acute eGFR changes with and without the treatment.

Notably, irrespective of $\rho_{\epsilon }$ (ranging from 0 to 1), we found no evidence that larger than average eGFR decline indicates a reduction in SGLT-2i’s long-term benefits. In fact, for higher $\rho_{\epsilon }$ values, our findings suggest a potential benefit associated with more substantial eGFR declines, suggesting that such a decline might signify a protective mechanism contributing to long-term benefit.

It is a natural human response to attribute changes in a biomarker after starting a new treatment to the treatment itself. This tendency is reflected in the common use of the expression “treatment response” to describe changes in biomarkers and other outcomes observed soon after treatment initiation. Similarly, clinicians and patients may assume that acute eGFR declines reflect effects of the treatment. However, as is evident from the substantial variation in acute eGFR changes in the placebo groups of the studies in our analysis (see Fig 2), where no new treatment was initiated, eGFR changes following treatment initiation must reflect multiple factors. These include measurement error and underlying variability in disease progression in addition to any true variation in the acute treatment effect. Distinguishing acute changes from actual causal acute effects is challenging because acute effects represent a comparison between outcomes with and without treatment, and such comparisons are unobservable. This limitation also prevents empirical determination of the sensitivity parameter $\rho_{\epsilon }$. Assumptions regarding the value of $\rho_{\epsilon }$ belong to the class of so called “cross-world” assumptions which are recognized as difficult to justify in causal inference.

As illustrated in Fig 1, the value of $\rho_{\epsilon }$ (or ρ) quantifies the amount of heterogeneity in the acute effect. Values close to 1 indicate that SGLT-2i’s produce uniform acute eGFR reduction across patients, implying that essentially all the variation in acute eGFR changes following SGLT-2i initiation are unrelated to the treatment. In contrast, values close to 0 imply substantial heterogeneity in the acute effect, implying that variation in acute effects could meaningfully contribute to observed variation in early eGFR changes after starting the treatment. As illustrated in Fig 1, the uncertainty in the value of $\rho_{\epsilon }$ has implications for the treatment effect on longer term clinical endpoints. In settings where larger acute eGFR declines are associated with increased risk of clinical events, large negative acute eGFR changes on the treatment could signify an attenuation or reversal in an overall beneficial effect if $\rho_{\epsilon }$ is close to 0, but not if $\rho_{\epsilon }$ is close to 1.

We observed only a negligible increase in the variability of the acute eGFR changes in the SLGT-2i groups compared to placebo in the studies of our analysis. This could suggest that any heterogeneity in the acute effect of SGLT-2i must have been small, consistent with a large $\rho_{\epsilon }$ (see Fig 2). However, similar variability in treatment and control groups could still occur under a heterogeneous acute effect if the acute effect were inversely correlated with acute eGFR changes with placebo. A stronger argument supporting a large $\rho_{\epsilon }$ is provided by noting that smaller $\rho_{\epsilon }$ and the consequent higher heterogeneity in the acute effect would imply that a substantial proportion of the study population must experience substantial positive acute effects (see the Panel 3 of Fig 1 and Table 3). We are unaware of any mechanism that could lead to positive acute effects greater than 1 ml/min/1.73 m^2^ in more than a very small fraction of patients. This suggests a large value for $\rho_{\epsilon }$, probably greater than 0.8.

Ultimately, across the entire spectrum of possible values for $\rho_{\epsilon }$, our data confirm that the observed initial eGFR change does not signal a reduction in the expected benefits for kidney failure or all-cause mortality. Further, for the higher and more plausible values of $\rho_{\epsilon }$, we observed a trend suggesting that more substantial acute declines may signify a protective hemodynamic mechanism leading to enhanced long-term preservation of kidney function, although the 95% confidence intervals for the treatment by acute eGFR change interaction effects indicated that this trend did not reach formal statistical significance. Overall, our results suggest that the magnitude of the acute eGFR change should not be viewed as a signal to de-escalate therapy, but rather as an expected physiologic response to treatment that is compatible with significant long-term clinical benefit.

The demonstration that acute eGFR declines do not diminish the long-term protective benefits of SGLT-2i has immediate implications for clinical decision making and patient communication. Clinicians should proactively counsel patients that an initial decline in estimated kidney function (via eGFR) commonly up to 20% within the first month of treatment, represents an expected and generally benign effect that reflects some combination of the expected variation that would have occurred without the treatment or consistent benign acute effects resulting from reduced intra-glomerular pressure. Because our analysis indicates that the substantial long-term relative risk reduction for kidney failure persists even in patients experiencing such acute declines, treatment should not be prematurely discontinued based solely on these early biomarker fluctuations. By utilizing a principal stratification framework, our methodology helps differentiate drug-induced hemodynamic changes from underlying disease progression, providing reassurance that even larger-than-average acute declines do not signal a reduction in therapeutic efficacy. Ultimately, these findings provide an evidence-based framework for clinicians to initiate SGLT-2i with confidence across a broad range of patient profiles, shifting the focus of clinical monitoring from transient early eGFR changes to the sustained prevention of kidney failure and mortality.

A potential limitation of our analysis is its reliance on the assumption that no unmeasured factor jointly influences both the acute effect and CKD progression under placebo. While we adjust for known confounders, which are strong predictors of CKD progression, it remains possible that an unobserved variable could introduce bias. Although we cannot entirely rule out this possibility, the confounders included in our analysis are well-established determinants of CKD progression. A further conceptual limitation is that the principal stratification approach used in this paper expresses the plausible range of treatment effects for clinical endpoints as a function of ΔeGFR, which can be observed only 1 month after the treatment has already been initiated. Because ΔeGFR is not known at the time of treatment initiation, it is not possible, in general, to use ΔeGFR to guide decisions regarding treatment initiation without additional assumptions. However, in this case, we saw no evidence for an adverse effect of SGLT-2i treatment across the full range of the ΔeGFR distribution, providing reassurance of no adverse effect of the treatment irrespective of any plausible eGFR declines that may subsequently be observed. A further limitation of the current analysis is that it does not explicitly model the joint relationship between acute eGFR changes and early changes in albuminuria, such as the albumin-to-creatinine ratio (ACR). Reductions in albuminuria are a hallmark of SGLT-2i therapy and are strongly associated with long-term kidney protection. Biologically, the acute eGFR decline and the reduction in proteinuria are often coupled, as both are consequences of the treatment’s ability to reduce intraglomerular pressure through tubuloglomerular feedback. By focusing solely on the eGFR decline, our study demonstrates that this specific biomarker change does not indicate a loss of benefit, even without accounting for concurrent ACR improvements. However, incorporating acute changes in ACR into our principal stratification framework would likely provide deeper mechanistic insights. For instance, it would be valuable to determine if the long-term benefit of SGLT-2i is even more pronounced in patients who experience both a significant acute eGFR decline and a substantial reduction in albuminuria.

Future research could extend our approach by considering the eGFR slope following the 1-month acute effect as the outcome, providing a more specific assessment of long-term kidney function decline beyond the initial acute effects. Building on the need for deeper mechanistic insights, our framework could be adapted to a multi-biomarker approach that incorporates the albumin-to-creatinine ratio, a key indicator of kidney disease progression and treatment response. Exploring the joint interplay between acute changes in ACR and eGFR would allow for a more granular assessment of long-term outcomes, potentially refining patient stratification and further distinguishing benign hemodynamic shifts from sustained renal protection. Beyond kidney disease, the methodology used in this report could be extended to other disease settings where early biomarker changes are predictive of long-term outcomes.

In conclusion, our analysis shows no evidence that a larger eGFR decline diminishes SGLT-2i’s benefits. For higher $\rho_{\epsilon }$ values, a more significant acute decline might even be associated with greater treatment efficacy.

## Supporting information

S1 FigEstimated interaction effect on kidney failure at 2 years.The figure displays the difference in the estimated 2-year risk reduction in kidney failure given the observed ΔeGFR after starting SGLT-2i and the 2-year risk reduction in kidney failure if ΔeGFR = 0 across different $\rho_{\epsilon }$. The ΔeGFR values are expressed as % changes.(DOCX)

S2 FigImplications of ΔeGFR on the estimated effect of SGLT-2i on the risk of death prior to kidney failure by 2 years.The figure displays the implications of the observed acute eGFR change (ΔeGFR) after starting SGLT-2i on the estimated effect of SGLT-2i on the risk of death prior to kidney failure within two years for alternative $\rho_{\epsilon }$. The analysis adjusts for baseline eGFR, log-transformed proteinuria, age, systolic blood pressure (SBP), sex and diabetes status. The relationship between ΔeGFR and death (with kidney failure as a competing event) was modeled using a restricted cubic spline with four knots. **The y-axis** shows the 2-year reduction in risk of death prior to kidney failure for patients treated with SGLT-2i compared to those without, using the steps outlined in Algorithm 1. **The bottom axis** represents the observed ΔeGFR after starting SGLT-2i, expressed as % change from baseline. **The top axis** indicates the corresponding estimated mean ΔeGFR with the placebo under the assumed value of $\rho_{\epsilon }$. **The steel blue line** shows the estimated reduction in the risk of death prior to kidney failure under the assumed $\rho_{\epsilon }$ given the observed ΔeGFR following SGLT-2i initiation, with 95% pointwise confidence intervals.(DOCX)

S3 FigEstimated interaction effect on death prior to kidney failure at 2 years.The figure displays the difference in the estimated 2-year risk reduction in death prior to kidney failure given the observed ΔeGFR after starting SGLT-2i and the 2-year risk reduction in death prior to kidney failure if ΔeGFR = 0 across different $\rho_{\epsilon }$. The ΔeGFR values are expressed as % changes.(DOCX)

S4 FigImplications of ΔeGFR on the estimated effect of SGLT-2i on the composite of kidney failure or death by 2 years.The figure displays the implications of the observed acute eGFR change (ΔeGFR) after starting SGLT-2i on the estimated effect of SGLT-2i on the risk of kidney failure or death within two years for alternative $\rho_{\epsilon }$. The analysis adjusts for baseline eGFR, log-transformed proteinuria, age, systolic blood pressure (SBP), sex and diabetes status. The relationship between ΔeGFR and the composite of kidney failure or death was modeled using a restricted cubic spline with four knots. **The y-axis** shows the 2-year reduction in risk of kidney failure or death for patients treated with SGLT-2i compared to those without, using the steps outlined in Algorithm 1. **The bottom axis** represents the observed ΔeGFR after starting SGLT-2i, expressed as % change from baseline. **The top axis** indicates the corresponding estimated mean ΔeGFR with the placebo under the assumed value of $\rho_{\epsilon }$. **The steel blue line** shows the estimated reduction in the risk of kidney failure or death under the assumed $\rho_{\epsilon }$ given the observed ΔeGFR following SGLT-2i initiation, with 95% pointwise confidence intervals.(DOCX)

S5 FigEstimated interaction effect on the composite of kidney failure or death at 2 years.The figure displays the difference in the estimated 2-year risk reduction in the composite of kidney failure or death given the observed ΔeGFR after starting SGLT-2i and the 2-year risk reduction in of kidney failure or death if ΔeGFR = 0 across different $\rho_{\epsilon }$. The ΔeGFR values are expressed as % changes.(DOCX)
