# Supplementary material for: Implications of acute change in estimated Glomerular Filtration Rate (eGFR) for the effect of sodium-glucose cotransporter-2 inhibitors (SGLT-2i) on long-term endpoints
Source: PLoS One. 2026 Apr 29;21(4):e0347741. doi: 10.1371/journal.pone.0347741 (PMC13128134; doi:10.1371/journal.pone.0347741)
Supplement: S3 Fig — The figure displays the difference in the estimated 2-year risk reduction in death prior to kidney failure given the observed ΔeGFR after starting SGLT-2i and the 2-year risk reduction in death prior to kidney failure if ΔeGFR = 0 across different ρϵ. The ΔeGFR values are expressed as % changes. (DOCX) [file pone.0347741.s003.docx]

**Supplementary S3 Fig. Estimated interaction effect on death prior to kidney failure at 2 years.** The figure displays the difference in the estimated 2-year risk reduction in death prior to kidney failure given the observed $\Delta eGFR$ after starting SGLT-2i and the 2-year risk reduction in death prior to kidney failure if $\Delta eGFR=0$ across different $\rho_{\epsilon}$. The $\Delta eGFR$ values are expressed as % changes.
