# Supplementary material for: Implications of acute change in estimated Glomerular Filtration Rate (eGFR) for the effect of sodium-glucose cotransporter-2 inhibitors (SGLT-2i) on long-term endpoints
Source: PLoS One. 2026 Apr 29;21(4):e0347741. doi: 10.1371/journal.pone.0347741 (PMC13128134; doi:10.1371/journal.pone.0347741)
Supplement: S4 Fig — The figure displays the implications of the observed acute eGFR change (ΔeGFR) after starting SGLT-2i on the estimated effect of SGLT-2i on the risk of kidney failure or death within two years for alternative ρϵ. The analysis adjusts for baseline eGFR, log-transformed proteinuria, age, systolic blood pressure (SBP), sex and diabetes status. The relationship between ΔeGFR and the composite of kidney failure or death was modeled using a restricted cubic spline with four knots. The y-axis shows the 2-year reduction in risk of kidney failure or death for patients treated with SGLT-2i compared to those without, using the steps outlined in Algorithm 1. The bottom axis represents the observed ΔeGFR after starting SGLT-2i, expressed as % change from baseline. The top axis indicates the corresponding estimated mean ΔeGFR with the placebo under the assumed value of ρϵ. The steel blue line shows the estimated reduction in the risk of kidney failure or death under the assumed ρϵ given the observed ΔeGFR following SGLT-2i initiation, with 95% pointwise confidence intervals. (DOCX) [file pone.0347741.s004.docx]

**Supplementary S4 Fig. Implications of** $\boldsymbol{\Delta}\boldsymbol{eGFR}$ **on the estimated effect of SGLT-2i on the composite of kidney failure or death by 2 years.** The figure displays the implications of the observed acute eGFR change ($\Delta eGFR$) after starting SGLT-2i on the estimated effect of SGLT-2i on the risk of kidney failure or death within two years for alternative $\rho_{\epsilon}$. The analysis adjusts for baseline eGFR, log-transformed proteinuria, age, systolic blood pressure (SBP), sex and diabetes status. The relationship between $\Delta eGFR$ and death (with kidney failure as a competing event) was modeled using a restricted cubic spline with four knots.

**The y-axis** shows the 2-year reduction in risk of kidney failure or death for patients treated with SGLT-2i compared to those without, using the steps outlined in Algorithm 1.

**The bottom axis** represents the observed $\Delta eGFR$ after starting SGLT-2i, expressed as % change from baseline.

**The top axis** indicates the corresponding estimated mean $\Delta eGFR$ with the placebo under the assumed value of $\rho_{\epsilon}$.

**The steel blue line** shows the estimated reduction in the risk of kidney failure or death under the assumed $\rho_{\epsilon}$ given the observed $\Delta eGFR$ following SGLT-2i initiation, with 95% pointwise confidence intervals.
